# Genome-Wide association analysis of phenotypic traits in Bambara groundnut under drought-stressed and non-stressed conditions based on DArTseq SNP

**DOI:** 10.3389/fpls.2023.1104417

**Published:** 2023-02-14

**Authors:** Kafilat Abiodun Odesola, Odunayo Joseph Olawuyi, Rajneesh Paliwal, Olaniyi Ajewole Oyatomi, Michael T. Abberton

**Affiliations:** ^1^ Department of Biological Sciences, Bells University of Technology, Sango Otta, Ogun State, Nigeria; ^2^ Genetic Resources Centre, International Institute of Tropical Agriculture, Ibadan, Oyo State, Nigeria; ^3^ Department of Botany, University of Ibadan, Ibadan, Oyo State, Nigeria

**Keywords:** accessions, underutilized, chromosome, drought, snps, DArTseq

## Abstract

**Introduction:**

Bambara groundnut (BG) (Vigna subterranea [L.] Verdc) is an indigenous, resilient, but underutilized leguminous crop that occurs mostly as genetically heterogeneous landraces with limited information on the drought tolerant attributes. This study elucidates the associations between sequencing-based diversity array technology (DArTseq) and phenotypic character as well as differing indices related to drought tolerance in one hundred accessions of Bambara groundnut.

**Methods:**

The field experiments were conducted at IITA research stations in Kano and Ibadan between 2016 and 2018 planting seasons. The experiments were arranged in randomised complete block design with three replications, under the different water regimes. The phenotypic traits evaluated was further to construct the dendrogram. Genome-wide association mapping was conducted based on 5927 DArTs loci with < 20% missing data.

**Results and Discussions:**

The genome wide association study predicted drought tolerance in Bambara accessions for geometric mean productivity (GMP) and stress tolerance index (STI). TVSu-423 had the highest GMP and STI values (28.50, 2.40), while TVSu-2017 had the lowest at GMP (1.74) and STI (0.01) respectively. The relative water content (%) was significantly higher for accessions; TVSu-266 (60.35, 61.49), TVSu-2 (58.29, 53.94), and TVSu-411 (55.17, 58.92) in 2016/2017 and 2017/2018, respectively. The phenotypic characters studied delineated the accessions into two major clusters and five distinct sub-clusters, indicating variations across all the geographical locations. The 5,927 DArTseq genomic markers in association with STI further grouped the 100 accessions into two main clusters. TVSu-1897 from Botswana (Southern Africa) was in the first cluster, while the remaining 99 accessions from Western, Central, and Eastern Africa made up the second cluster. The eight significant Quantitative Trait Loci (QTLs) (24346377|F|0-22:A>G-22:A>G, 24384105|F|0-56:A>G33 :A> G, 24385643|F|0-53:G>C-53:G>C, 24385696|F|0-43:A>G-43:A>G, 4177257|F|0-44:A>T-44:A>T, 4182070|F|0-66:G>A-66:G>A, 4183483|F|0-24:G>A-24:G>A, 4183904|F|0-11:C>T-11:C>T) identified with Bonferroni threshold was in association with STI, indicative of variations under the drought-stressed condition. The observation of consistent SNPs in the 2016 and 2017 planting seasons, as well as in combination with the 2016 and 2017 planting seasons, led to the designation of these QTLs as significant. The drought selected accessions could form basis for hybridization breeding. The identified quantitative trait loci could be useful in marker-assisted selection in drought molecular breeding programs.

## Introduction

Given its unique resilience attributes, Bambara groundnut (BG) (*Vigna subterranea*) belongs to the group of underutilized crops presently being promoted as climate-smart crops (Feldman et al., 2019). Underutilized plants provide several impressive health benefits. They are also better adapted to marginal lands and biotic and abiotic stress conditions. They can contribute significantly to the diversification and resilience of agroecosystems (Dogan et al., 2014; Saran et al., 2021; Bozhuyuk, 2022). Throughout much of sub-Saharan Africa, it is a widely cultivated grain legume (Directorate Plant Production, 2011; Ahmad, 2013). BG is a highly self-fertilizing crop with a rather variable yield owing to its inconsistency in its response to different environmental circumstances, among which is drought stress (Bamshaiye et al., 2011). Drought tolerance is a quantitative trait with a lot of complex genetic and phenotypic control and gradual improvement (Sinclair, 2011). Prior research work on quantitative traits was based on the identification of single gene effects on phenotype (Orgogozo et al., 2015), until the recent advances in genomics and molecular biology improved the identification of candidate genes and quantitative trait loci (QTLs) through the simultaneous dissection of these complex traits using agronomic traits, gene region and association analysis (Jha et al., 2022; Wang et al., 2022).

The genome-wide association study (GWAS) is the commonest approach to understanding the association between the complexities controlling most agronomic traits of interest to their genetic bases. It relies on the association of several representative markers and a huge genetically diverse population of organisms. It finds the linkage disequilibrium between the phenotypic trait and the genetic markers (Korte and Farlow, 2013) without any prior knowledge of the level of the kinship of the organism. This mapping approach makes use of the occurrence of rare alternative forms of a gene on a particular chromosome at different gene locations to identify a common site of interest for any genetic relationship (Flint-Garcia et al., 2003; Mackay and Powell, 2007). Another benefit of association mapping for the study of a quantitative trait is that it is highly useful for researching species that are hard to cross or clone or that take a long time to reproduce (Nordborg and Weigel, 2008). Overall GWAS helps find markers, genes, or QTLs associated with phenotypic traits that can conveniently be used for gene introgression, gene discovery, or marker-assisted breeding.

The basic DNA-based marker systems used in mapping the most complex traits in different crop species include simple sequence repeat (SSR) and Amplified fragment length polymorphism (AFLP), which are mostly gel-based markers and are limited in their ability to perform rapid analysis on a large number of marker loci. Single Nucleotide Polymorphism (SNP) and the Diversity Arrays Technology (DArT) marker system are the most recent and better mapping tools, and they become more robust when used together (Nadeem et al., 2018). Diversity Array Technology sequencing (DarTseq) is one novel platform under the next-generation sequencing (NGS) platform developed to perform a simultaneous Single Nucleotide (SNP) discovery of unique nucleotides among a population, particularly for non-model germplasm sets (Kilian et al., 2012; Raman et al., 2014). This method for studying genetic diversity is based on the reduction of complexity through the application of restriction enzymes that target gene-rich areas. It, thus, thoroughly covers the genome using DarTseqTM technology to provide a high-density genetic map that increases the likelihood of finding QTL (Thudi et al., 2011). The combination of SNPs and the NGS reported for other crops of interest had been identified to bring uniqueness to the identification of genetic variants (SNPs), phylogenetics, germplasm assessment, and population structure (Grzebelus et al., 2014). A few of the grain crops on which DarT markers have previously been used include pigeon pea (Yang et al., 2011), soybean (Vu et al., 2015), and common bean, (Valdisser et al., 2017). Given the abundance of SNPs, the uniqueness of the NGS platform, and the complexity of drought trait, this study, therefore, aims to identify marker-trait association of some phenotypic traits of Bambara groundnut using the representative DarTseq to discover relevant QTLs for possible future breeding work.

The specific objectives of the present study are:

(ii) To evaluate the performance of DarTseq method-derived markers in Bambara groundnut,

(ii) Map QTLs/for drought tolerance in Bambara groundnut.

## Materials and methods

### Plant material and experimental design

For the study, 100 accessions of Bambara groundnut seeds from 12 African countries were collected from the International Institute of Tropical Agriculture (IITA), Ibadan, Nigeria. The 100 accessions (**Table 1**) were subjected to intense phenotyping of key traits related to drought. Six of these traits were finally selected after a thorough field experiment involving two planting seasons (2016/2017 and 2017/2018), two water regimes (well-watered (WW) and water-stressed (WS) conditions), and two different locations (Ibadan and Kano stations research fields of IITA). The trials were set up using a Randomized Complete Block Design (RCBD) with three replications. Each plot measured 1 m by 2.5 m and the distances between rows were 1.00 meters and 0.25 meters, respectively. The population was phenotyped for the number of days to 50% flowering (FLW), Visual scoring (VSL) at three weeks, at six weeks and at the termination of the experiment after the imposition of drought stress, leaf chlorophyll content (CHL), number of seeds per plot (SPL), weight of seeds per plot (WTPL), and grain yield per plot (GYLD). The data were further subjected to yield trait analysis using the eleven tolerant indices: Stress Susceptibility Index (SSI), Relative Drought Index (RDI), Stress Tolerance Index (STI), Geometric Mean Production (GMP), Tolerance Index (TOL), Mean Production (MP), Yield Index (YI), Drought Resistance Index (DI), Yield Stability Index (YSI), Stress Susceptibility Percentage Index (SSPI), and Modified Stress Tolerance (MSTI).

**Table 1:** List of the accessions with their countries of origin.

S/N	Acc Name	Country of origin	S/N	Acc Name	Country of origin
1	TVSu-188	Benin	51	TVSu-250	Gambia
2	TVSu-189	Benin	52	TVSu-251	Gambia
3	TVSu-193	Benin	53	TVSu-252	Gambia
4	TVSu-194	Benin	54	TVSu-1	Nigeria
5	TVSu-203	Benin	55	TVSu-2	Nigeria
6	TVSu-288	Benin	56	TVSu-4	Nigeria
7	TVSu-1092	Benin	57	TVSu-5	Nigeria
8	TVSu-1897	Botswana	58	TVSu-9	Nigeria
9	TVSu-85	Burkina Faso	59	TVSu-10	Nigeria
10	TVSu-86	Burkina Faso	60	TVSu-22	Nigeria
11	TVSu-292	Burkina Faso	61	TVSu-174	Nigeria
12	TVSu-1157	Burkina Faso	62	TVSu-182	Nigeria
13	TVSu-1161	Burkina Faso	63	TVSu-258	Nigeria
14	TVSu-1162	Burkina Faso	64	TVSu-265	Nigeria
15	TVSu-1164	Burkina Faso	65	TVSu-266	Nigeria
16	TVSu-1166	Burkina Faso	66	TVSu-282	Nigeria
17	TVSu-1175	Burkina Faso	67	TVSu-286	Nigeria
18	TVSu-2017	Burundi	68	TVSu-326	Nigeria
19	TVSu-2018	Burundi	69	TVSu-338	Nigeria
20	TVSu-395	Cameroon	70	TVSu-354	Nigeria
21	TVSu-399	Cameroon	71	TVSu-362	Nigeria
22	TVSu-409	Cameroon	72	TVSu-593	Nigeria
23	TVSu-411	Cameroon	73	TVSu-595	Nigeria
24	TVSu-416	Cameroon	74	TVSu-682	Zambia
25	TVSu-418	Cameroon	75	TVSu-687	Zambia
26	TVSu-421	Cameroon	76	TVSu-689	Zambia
27	TVSu-423	Cameroon	77	TVSu-690	Zambia
28	TVSu-434	Cameroon	78	TVSu-691	Zambia
29	TVSu-442	Cameroon	79	TVSu-692	Zambia
30	TVSu-445	Cameroon	80	TVSu-693	Zambia
31	TVSu-447	Cameroon	81	TVSu-699	Zambia
32	TVSu-448	Cameroon	82	TVSu-702	Zambia
33	TVSu-449	Cameroon	83	TVSu-713	Zambia
34	TVSu-459	Cameroon	84	TVSu-716	Zambia
35	TVSu-504	Cameroon	85	TVSu-719	Zambia
36	TVSu-1277	Central African Republic	86	TVSu-725	Zambia
37	TVSu-1278	Central African Republic	87	TVSu-731	Zambia
38	TVSu-1284	Central African Republic	88	TVSu-736	Zambia
39	TVSu-1285	Central African Republic	89	TVSu-745	Zambia
40	TVSu-1289	Central African Republic	90	TVSu-978	Zimbabwe
41	TVSu-1290	Central African Republic	91	TVSu-989	Zimbabwe
42	TVSu-1296	Central African Republic	92	TVSu-1011	Zimbabwe
43	TVSu-1309	Central African Republic	93	TVSu-1014	Zimbabwe
44	TVSu-1320	Central African Republic	94	TVSu-1015	Zimbabwe
45	TVSu-1373	Central African Republic	95	TVSu-1023	Zimbabwe
46	TVSu-1991	Congo	96	TVSu-1026	Zimbabwe
47	TVSu-115	Ivory Coast	97	TVSu-1034	Zimbabwe
48	TVSu-116	Ivory Coast	98	TVSu-1051	Zimbabwe
49	TVSu-118	Ivory Coast	99	TVSu-1056	Zimbabwe
50	TVSu-247	Gambia	100	TVSu-1078	Zimbabwe

TVSu, Tropical Vigna Subterranea.

### DNA extraction and quantification

The 100 accessions of Bambara groundnut, plus two as control, were raised in controlled environments at the IITA research field and leaf tissues were sampled for DNA extraction at 4 weeks after planting. Young leaf tissue weighing one gram was harvested, promptly frozen in liquid nitrogen, and then kept at 80°C. Using a typical cetyltrimethylammonium bromide (CTAB), chloroform, and isoamyl alcohol technique with a little modification by the addition of 20% Sodium Dodecyl Sulphate (SDS), genomic DNA was recovered from the frozen leaves (Doyle and Doyle, 1987). The quality of the DNA was performed with 0.8% agarose gel while purity checks at 260/230 and 260/280 nm absorbance ratios were done with Nanodrop spectrophotometer (ND2000 V3.5, NanoDrop Technologies, Inc.). Still using the Nanodrop spectrophotometer, a final adjustment of the DNA was performed to 100 ng/µl for subsequent DarT and SNP genotyping.

### Genotyping of individual samples using DarTseq technology

The 102 accessions of Bambara groundnut were forwarded to Diversity Arrays Technologies commercial service Ltd., Australia (www.diversityarrays.com) for individual genotyping with the HiSeq 2000 (Illumina) next-generation sequencer. After rigorous quality control and filtering with a call rate of 80%, marker reproducibility of 95%, and missing data of 20%, 5,927 SNPs were found to be polymorphic out of a total of 11,821 DarTseq markers generated for further analysis. Phenotypic data were subjected to analysis of variance (ANOVA) using the Statistical Analysis System (SAS 9.0) while the treatment means were all compared using the Duncan Multiple range test at a probability level of 0.05. To ascertain the association significance using the established threshold P<1.68× 10^−4^ (Li et al., 2016), the field data were subsequently subjected to the Best Linear Unbiased Prediction (BLUP) and correlated with the genetic sequence using the TASSEL molecular software version 5.2.23 for the Genome-Wide Association Study.

## Results

### Phenotypic traits evaluation

The result of mean square variance showed significant differences (P< 0.001) in the performances of the 100 Bambara groundnut accessions with regards to location, year water regime, and their interaction effects for all the studied traits. High and positive correlations were observed for all traits, however, no significant difference was recorded for the year effect on the 50% flowering rate while the second-order interactive effect of water and location on chlorophyll was also not significant. Analysis of variance further indicated significant differences in the chlorophyll content index among the accessions with respect to location, water treatment condition, and all other studied interactions except for water treatment/location combination (**Table 2**).

**Table 2:** Mean squares for the traits for 100 accessions of Bambara groundnut planted in Ibadan and Kano in the 2015/16 and 2016/17 planting seasons.

Source	DF	FLW	CHL	VSL	RWC	SPL	WTPL
ACCNS	99	708.34***	1038.03***	3.95***	3066.66***	4771.58***	810.47***
LOCATN	1	1950.49***	19084.83***	98.91***	879801.16***	725903.82***	158246.62***
YEAR	1	6876.45NS	41384.07***	725.49***	8825.63***	85697.04***	26723.03***
WTRT	1	369.72***	1604.99***	43.97***	3142.25**	434093.37***	66457.48***
WTRT*LOCATN	2	405.13**	10.89NS	20531.97***	114.98***	143303.4****	800.24***
LOCATN*YEAR	1	192.71***	7181.83***	1021.87***	34969.76***	114.98***	1586.89***
ACCNS*WTRT	99	18.24***	95.45NS	2.02**	609.23**	2554.66***	626.7***
ACCNS*LOCATN	99	126.76***	297.34***	3.14***	1448.56***	3922.05***	413.09***
ACCNS*YEAR	99	273.05***	432.67***	3.28***	916.01***	2631.76***	442.45***
ACCN*WTRT*LOCAT*YEAR	398	53.74NS	192.54***	1.86***	596.15**	0.95***	350.18***
POOLED ERROR	801	179.86***	412.22***	6.66***	2225.64***	4439.87***	807.97***

DF-Degree of freedom; FLW-Days to flowering; VSL-Visual Scoring; RWC-Relative water content; SPL-Number of seeds/plotWTPL-Weight of seeds/Plot; Levels of significance - (**P<-0.01%, ***P<0.001%.)

ACCNS-Accessions; WTRT-Water treatment; LOCATN-location; YEAR-Year.

ACCNS*WTRT–Accessions/water treatment interaction; ACCNS*LOCATN-Accession/location interaction.

ACCNS*WTRT-accession/water treatment interaction; ACCNS*YEAR-accession/year interaction.

WTRT*LOCATN–Water treatment by location interaction; LOCATN*YEAR-location/year interaction.

ACCN*WTRT*LOCAT*YEAR-Accession/water treatment/location/year interaction.

### SNPs polymorphism, heterozygosity, minor allele frequency

**Figure 1:**
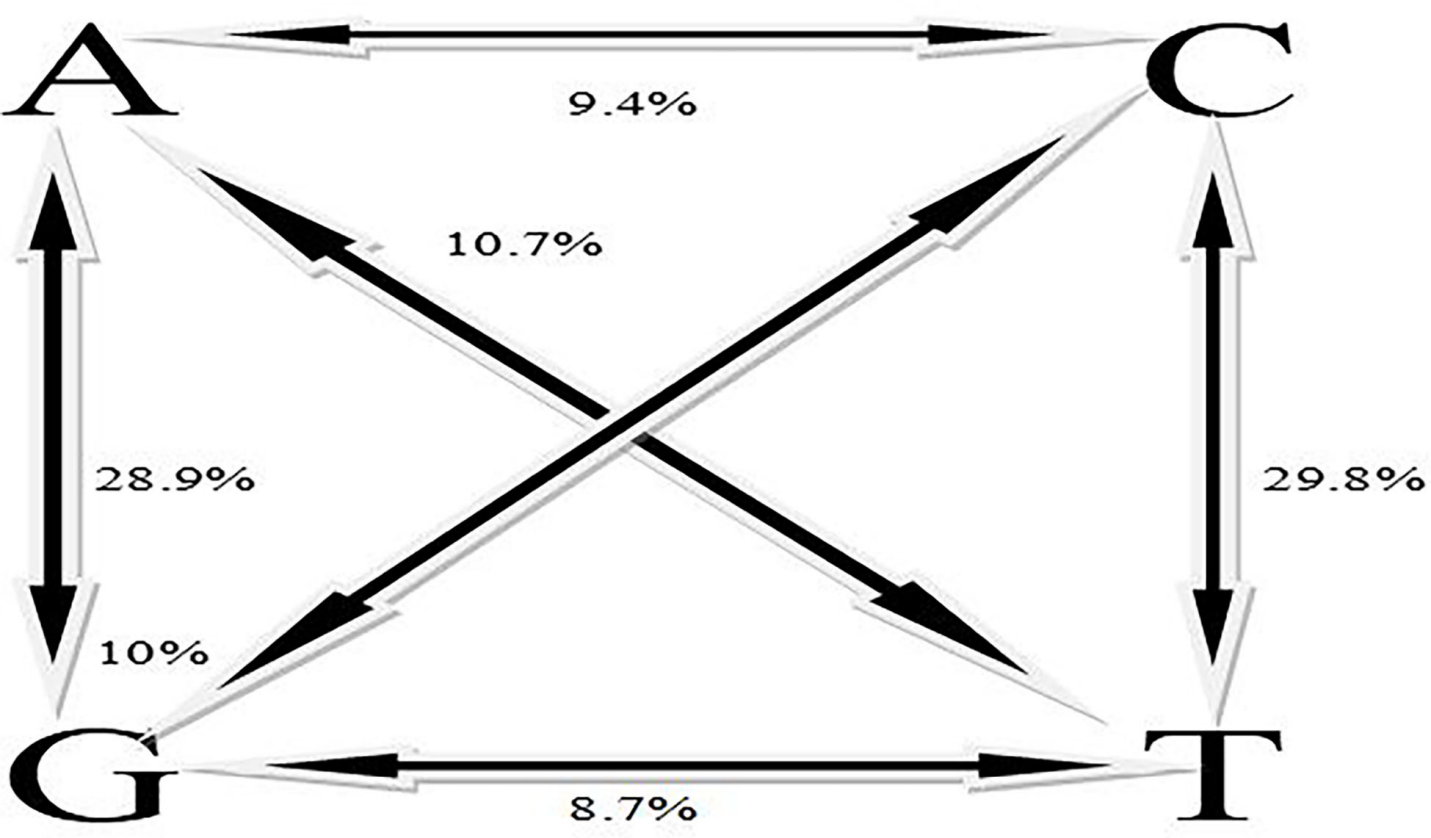
Percentage distribution of transitions and transversions among the SNPs.

**Figure 2:**
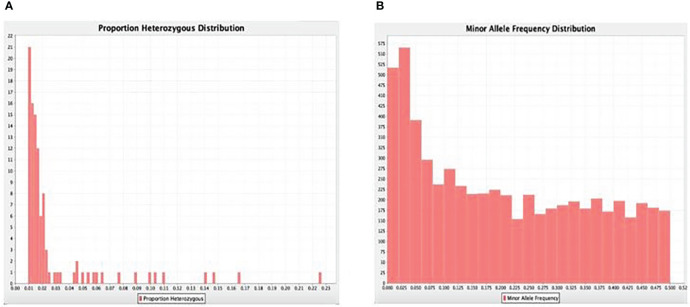
**(A)** Gene summary showing the frequency proportions of the heterozygosity frequency and **(B)** minor (rare) alleles.

A total number of 11,821 DarTseq markers were discovered and from these, 5,927 SNPs showed polymorphism and were subsequently retained for further analysis after thorough quality control and filtering were ascertained. Quality control was ensured at a consistent call rate ≥ 80%, while marker reproducibility was at ≥ 95%, and missing data ≤ 20%. The most frequently occurring polymorphism type was a cytosine to thymine combination (907; 15.3%), followed by guanine to adenine (871; 14.7%), with the least being observed with guanine to thymine at 4% and a proportion of 255 out of the whole 5927 SNPs (**Table 3**). The sequenced DNAs generated more transition (Ts) substitutions than transversion (Tv) substitutions at a percentage ratio of 58% to 39%, leading to an overall value of 1.52 for Ts/Tv (**Table 4**). Transitions C↔T and A↔G were represented with 29.8% and 28.9% of the total substitutions, respectively, while the four transversion classes occurrences were as displayed in **Figure 1**. The higher occurrence of transition showed that the SNPs occurrence is more in the coding region than in the non-coding region. Furthermore, the fraction of homozygous (G:G, C:C, A:A, and T:T) observations for individual SNPs were all in the percentage ratio of 0.5% to 0.8%. A range of 1% to 23% heterozygosity was observed among the 100 accessions, with an overall average of 0.06%. Of the accessions, 21 had 1% heterozygosity, with 67 of the accessions falling within the < 5% of recommended heterozygous rate for a self-pollinating plant. The remaining 12 accessions had heterozygous tendencies between 6% to 23%. The identified individual accession level of outcrossing evidenced the opportunity that could accrue from exploiting some of these accessions for further breeding purposes (**Figure 2A**). The set criteria for the minor allele frequency (MAF) were at <0.01 to show that the rare allele frequency was well distributed and that the 5,927 SNP markers generated by DArTseq came from distinct sequences. The majority of MAFs were well distributed (**Figure 2B**).

**Table 3:** Summary of genomic sequencing.

Alleles	Number	Proportion	Frequency
C	144338	0.23875	0.25002
G	139777	0.23121	0.24212
A	138614	0.22928	0.24011
T	138098	0.22843	0.23921
N	27250	0.04507	0.0472
Y	5637	0.00932	0.00976
R	4896	0.0081	0.00848
W	1852	0.00306	0.00321
M	1479	0.00245	0.00256
S	1418	0.00235	0.00246
K	1195	0.00198	0.00207
C:T	907	0.15303s
G:A	871	0.14695	
T:C	860	0.1451	
A:G	839	0.14156	
T:A	329	0.05551	
A:T	305	0.05146	
G:C	305	0.05146	
A:C	289	0.04876	
C:G	285	0.04809	
C:A	266	0.04488	
T:G	259	0.0437	
G:T	255	0.04302	
G:G	48	0.0081	
C:C	44	0.00742	
A:A	33	0.00557	
T:T	32	0.0054	
Total SNPs	5927		

C-Cytosine, G-Guanine, A-Adenine, T-Thymine, Y-pyrimidine (C or T), R-Purine (A or G),

S-Strong (G or C), N-Any nucleotide, W-(A or T), M-amino (A or C), K- Keto (G or T).

**Table 4:** Degree of transition and transversion identified using DarTseq.

Polymorphism Type	Allele	Number of allelic sites	Percentage of allelic sites	Total percentage
Transition	A↔G	1710	28.9%	
	C↔T	1767	29.8%	**3,477(58.7%)**
	A↔C	555	9.40%	
	A↔T	634	10.70%	
	G↔C	590	10.00%	
	G↔T	514	8.70%	**2,293(38.7%)**

### Population distribution

**Figure 3:**
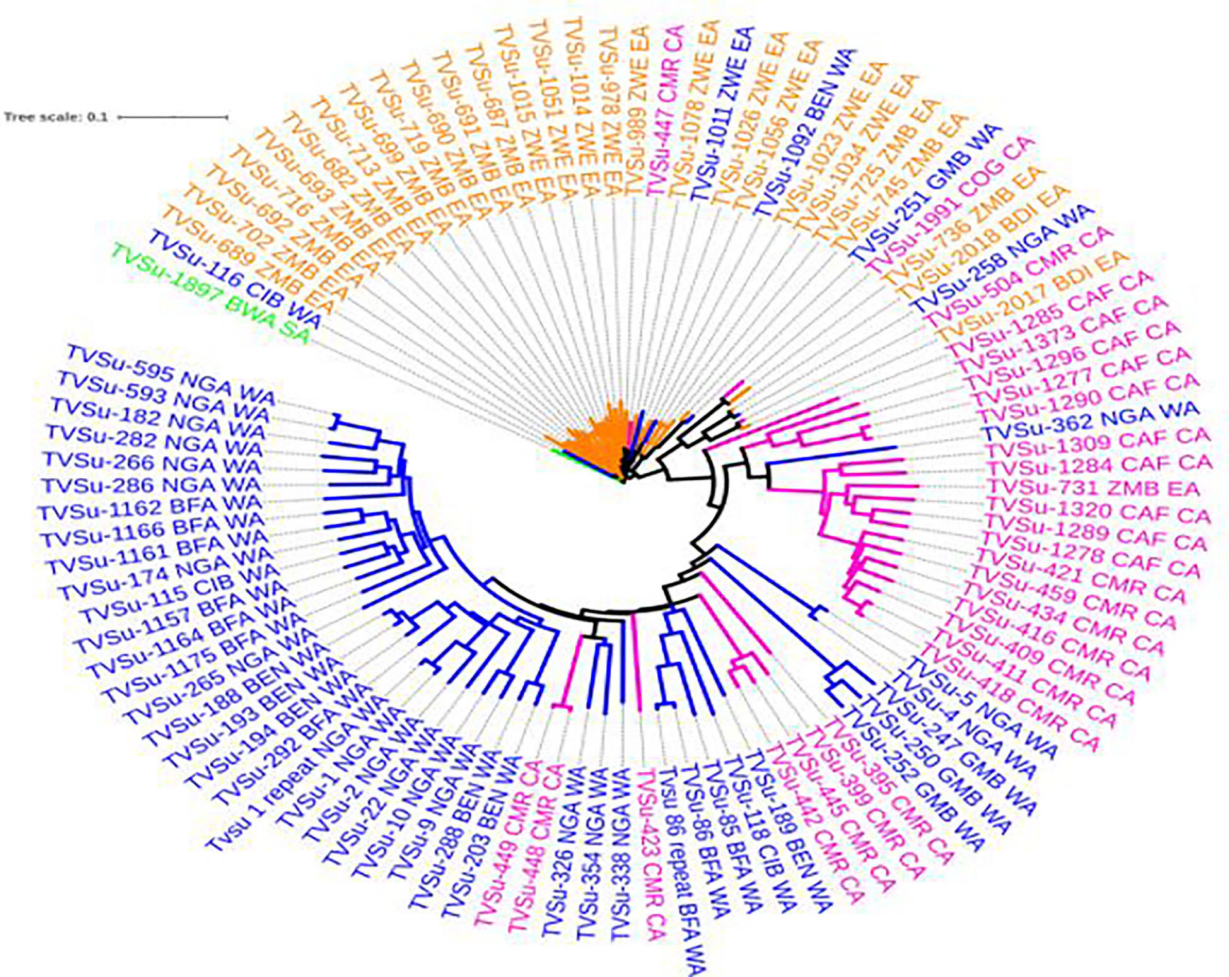
Phylogenetic relationship among Bambara groundnut accessions from 12 countries. Interactive tree of Life (ITOL) Letunic and Bork (2016) *Nucleic Acids Res*.

Neighbor-Joining Phylogeny was generated with the DArT markers, and these produced several sub-clusters of related accessions. Clustering was mostly based on agro-climatic areas and a possible similar genetic background. This was evident with an observed delineation into two main clusters, with the first cluster harboring just one accession sampled from South Africa. The remaining 99 accessions were within three sub-clusters, having accessions from West, Central, and East Africa all clustering together. Within the 44 West Africa accessions, there were approximately seven Central Africa accessions clustered within. The uniqueness of these seven accessions was in the fact that they all originated from Cameroon. While 28 selections were from Central Africa, the remaining 27 accessions were from East Africa. Accession TVSu-362 from West Africa was found among the bulk of the Central Africa accessions, while four different accessions from East Africa and one accession from West Africa were observed to have clustered among the 27 Central Africa accessions (**Figure 3**).

#### Principal coordinate analysis (PCA)

**Figure 4:**
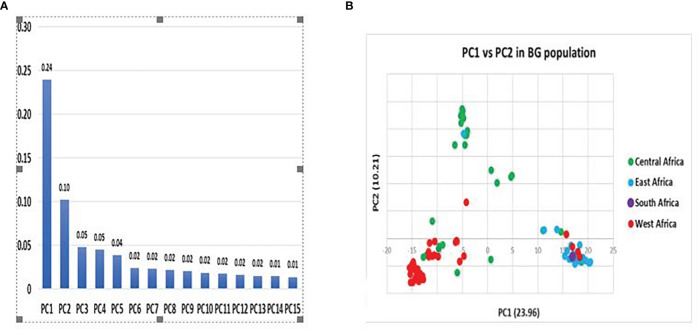
**(A)** Principal component contribution and **(B)** Principal coordinate analysis of total variation of Bambara accessions population based on DArTseq.

The principal coordinate analysis (PCA) was exploited to measure the observed variations based on the DArTseq markers. The PCA is explained by fifteen components with wide-ranging variations, as the first two principal coordinates explain the largest proportion of the genetic diversity. Of the total variations, 34 (34%) were accounted for by the PC1 while the remaining variations observed by the rest of the components were accounted for at just 2.82%. (**Figure 4A**). Furthermore, the PCA approach, which is mainly based on the genetic distance matrix and other data standardization, showed a consistent pattern with the phylogeny and grouped the 100 accessions of Bambara into three major clusters, similar to what was observed with the dendrogram. On the left side of the quadrant and colored red, are the accessions tagged to have originated from West Africa, with the few accessions colored green being known to have been from Central Africa. Likewise, on the right are accessions from East Africa with few accessions from Central Africa clustering together. In addition to the cluster is the accession originating from South Africa. (**Figure 4B**). In the dendrogram, the Southern Africa accessions occupied a main cluster delineating it from other accessions. The middle of the quadrant is occupied by accessions coming from Central Africa with some accessions coming from East Africa.

#### Marker-trait associations

**Figure 5:**
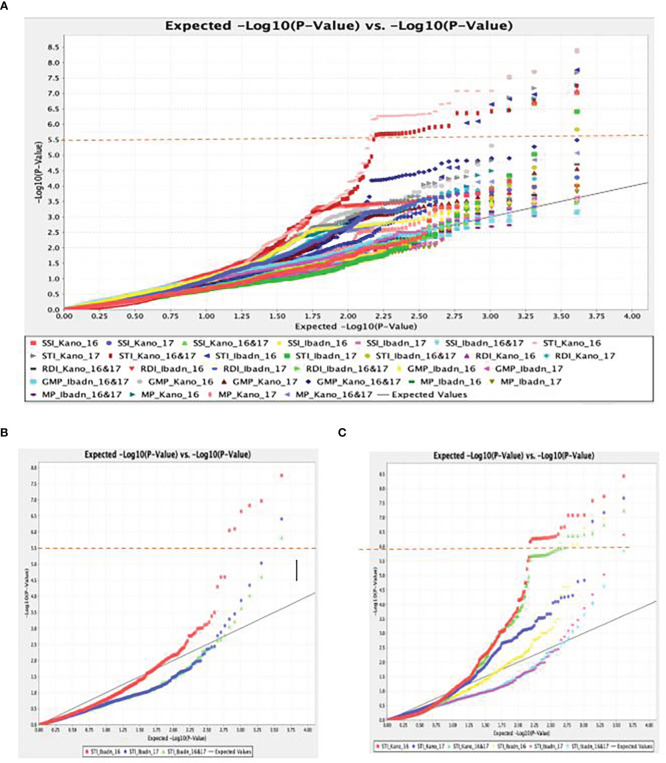
**(A)** Quantile–quantile (Q-Q) plot of a mixed linear model for Stress Tolerant Indices (STI) for drought studies in a panel of accessions of Bambara groundnut. **(B)** The p values of the SNPs and Quantile–quantile (Q-Q) plot of p values for stress-tolerant indices under the two water regimes in Ibadan. **(C)** The p values of the SNPs and Quantile–quantile (Q-Q) plot of p values for stress-tolerant indices under the two water regimes in Kano for the two years.

**Figure 6:**
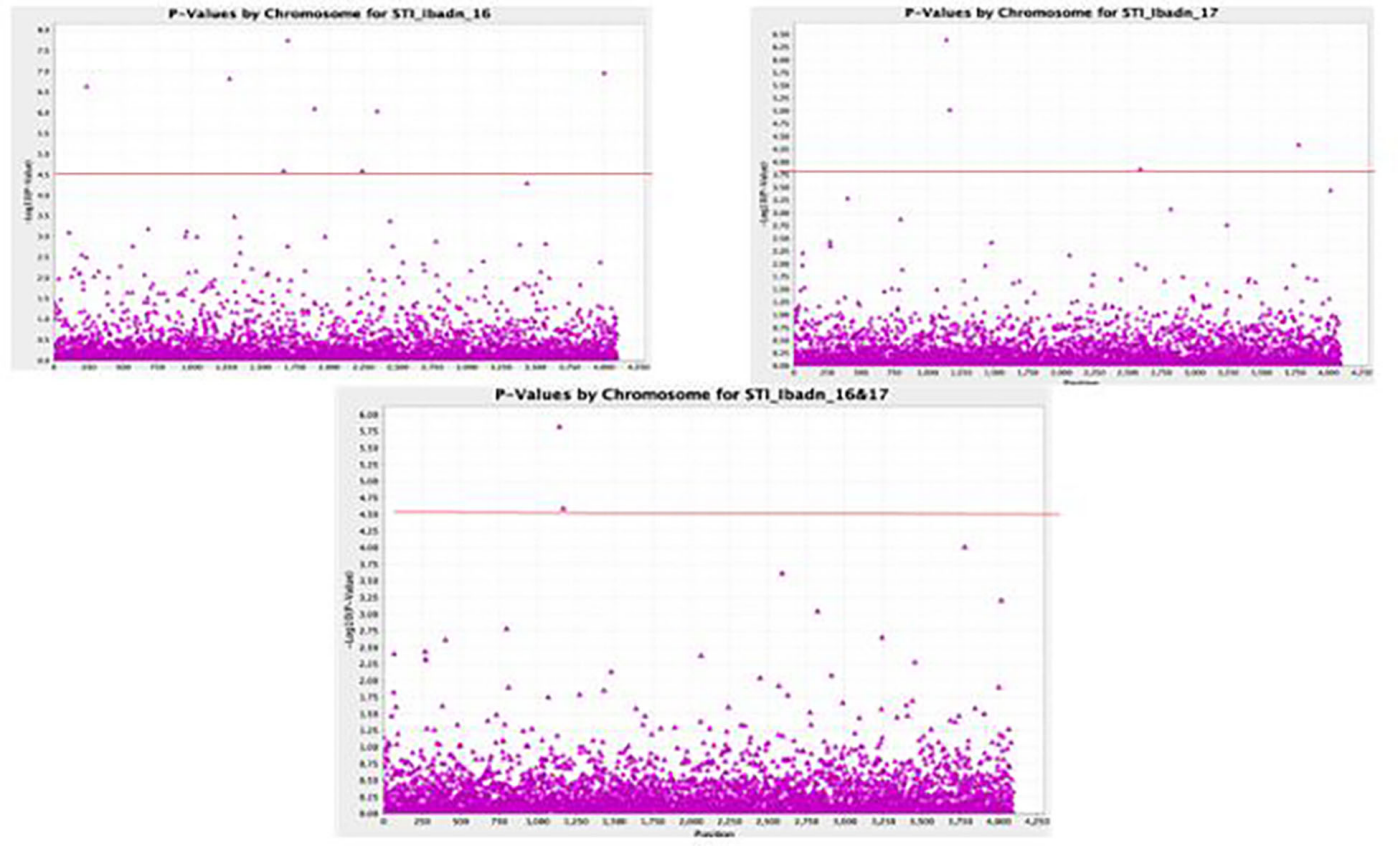
GWAS Manhattan Plot identifying the QTLs for Stress Tolerance Indices in Kano for two years.

A total of 11,083 “raw” SNPs with potential InDels were found; following careful filtering of SNPs, which excluded markers with more than 20% incomplete data and, thus, a read depth of >80% marker’s call rate, and minor allele frequency (MAF > 0.01) on the 102 lines, there was a yield of 5927 SNPs. Of the 5927 SNPs, 4095 sequences could not be definitively linked to any of the referred mung bean genomes, while the remaining 1595 SNPs were evenly scattered across the whole “Mung bean chromosome”. Chromosomes 5 and 7 contained the most SNPs (203 SNPs), followed by Chromosome 8 (199 SNPs), Chromosome 1 (158 SNPs), Chromosome 6 (148 SNPs), Chromosome 11 (134 SNPs), Chromosome 10 (132 SNPs), Chromosome 3 (120 SNPs), Chromosome 2 (119 SNPs), and Chromosome 9 (98 SNPs). Approximately 5% of markers were mapped to scaffolds that are still unrelated to a chromosome, making up a tiny portion of the total. The (-log p> 5.5, a= 1) threshold was taken as the cutoff point for significant detection of the marker-trait relationship, as shown in the first Quantile-Quantile figure (QQ plot) (**Figure 5A**). The different colors (blue, red, and pink) were observed to be aligned over this threshold, and traits associated with these colors were identified to be STI Kano 16, STI Kano 16 & 17, and STI Ibadan 16. Delineating the QQ plot on a location basis gave a clearer picture of correlation at the same threshold with the distinct red color observed for the Ibadan location and the colors red, green, and blue above the threshold relating the significance of associated trait for the individual years and the combination of the years for the two locations of Ibadan and Kano (**Figures 5B, C**). The total 5,927 SNPs identified thus fulfilled these conditions, and as such, this number of SNPs was used to perform the association analysis with the stress tolerance indices. This significant marker was generated using the mixed linear model (MLM) association analysis and it was only detected for Stress Tolerance Index (STI), Geometric mean productivity (GMP), Relative Drought Index, and Mean Productivity (MP). No significant markers were detected for the other drought indices, namely, Tolerance index (TOL), Stress Susceptibility Index, Drought Index (DI), Yield index, Yield stability index (YSI), Modified stress tolerance index (k1STI and k2STI), and Susceptibility percentage index (SSPI) under the different water regimes for the two years of planting and the two locations generated data. The significant association between the phenotypic traits and the SNPs marker is further displayed with the Manhattan plots (**Figure 6**). The Manhattan plot is showing scattered outliers that are situated on the upper part of the plot at a surpassed threshold of 0.5. The genome-wide significance threshold is indicated by the red line and the SNPs associated with the trait of interest are highlighted in purple dots above the threshold. The SNPs were significantly associated with the stress-tolerant indices for 2016, 2017, and a combination of the 2016 and 2017 years of planting of the accession in the Kano state. Eight significant SNPs were identified based on the mixed linear model (MLM) association analysis. Worthy of note was the fact that the identified SNPs were unique for the Kano location in association with the Stress Tolerance Indices. The markers range for the expression of the phenotypic variation (R2) was between 0.19% and 0.31%, which is within the typical P value for an SNP to be significant. The eight SNPs, thus, were significant given that all p values increased exponentially beyond the standard adjustment threshold of p < 5 × 10−8 (**Table 5**).

**Table 5:** Summary of the eight diagnostic SNPs.

ID No	Trait	Marker	P-value	MarkerR2
8	STI_Kano_16		5.41E-07	0.34844
**9**	**STI_Kano_16&17**	**24346377|F|0-22:A>G-22:A>G**	**2.06E-06**	**0.30767**
10	STI_Kano_17		9.63E-04	0.15484
31	STI_Kano_16		7.11E-05	0.21889
**32**	**STI_Kano_16&17**	**24384105|F|0-56:A>G-56:A>G**	**2.57E-04**	**0.18418**
33	STI_Kano_17		3.59E-04	0.17897
63	STI_Kano_16		1.82E-05	0.25324
**64**	**STI_Kano_16&17**	**24385643|F|0-53:G>C-53:G>C**	**1.24E-04**	**0.20176**
65	STI_Kano_17		8.07E-05	0.21605
67	STI_Kano_16		1.84E-05	0.25299
**68**	**STI_Kano_16&17**	**24385696|F|0-43:A>G-43:A>G**	**1.25E-04**	**0.20175**
69	STI_Kano_17		8.91E-05	0.21353
136	STI_Kano_16		4.74E-07	0.35216
**137**	**STI_Kano_16&17**	**4177257|F|0-44:A>T-44:A>T**	**2.00E-06**	**0.30857**
138	STI_Kano_17		7.08E-04	0.16227
182	STI_Kano_16		4.70E-07	0.35242
**183**	**STI_Kano_16&17**	**4182070|F|0-66:G>A-66:G>A**	**1.77E-06**	**0.31191**
184	STI_Kano_17		7.44E-04	0.16107
201	STI_Kano_16		3.64E-05	0.23528
**202**	**STI_Kano_16&17**	**4183483|F|0-24:G>A-24:G>A**	**1.07E-04**	**0.2055**
203	STI_Kano_17		6.81E-04	0.1632
209	STI_Kano_16		5.35E-07	0.34985
**210**	**STI_Kano_16&17**	**4183904|F|0-11:C>T-11:C>T**	**2.22E-06**	**0.30628**
211	STI_Kano_17		7.06E-04	0.16268

## Discussion

Identifying the key genetic underpinnings of complex features is very critical in organisms as it gives an accurate measure of its diversity with respect to any trait of interest. This study, thus, explored the association of the genomic region and the yield-related traits of a diverse population of Bambara groundnut for drought tolerance using the New Generation Sequencing (NGS) platform. The application of the NGS platform has been described to give in-depth knowledge of an organism’s genome compared to the regular markers (AFLP, RAPD, ISSR, SRAP, and SSR) application. A wide range of performances within and across environments for most of the studied drought-related traits observed with high estimates of values as well as significant and positive correlations among the 100 accessions of Bambara groundnut was obtained. For an effective association between the phenotype and genotype traits of interest through the GWAS approach, the population size, trait of interest, marker density, and phenotypic evaluation were all considered to be some of the key factors to variations. The high population size gave allowance for the production of a higher number of markers and, thus, a strong verdict of the power of the QTLs. This study used 100 accessions and this agrees with what was observed on GWAS by Upadhyaya et al. (2013), Dang et al. (2016), and Zhou et al. (2017), who all documented a population range of 100-300 for an effective GWAS analysis. Although these large numbers of accessions can sometimes be hindered by factors, such as the cost of analysis, availability of the platform for the analysis, as well as the plant species itself, and the specific traits of interest. Research has been conducted on the effective utilization of DarTseq for screening, which includes drought tolerance in wheat (Mwadzingeni et al., 2017), barley (Wójcik-Jagła et al., 2018), and rust resistance in *Pisum fulvum* (Barilli et al., 2018), as well as the comparative genome analysis in fish (Shams et al., 2019) amongst others.

To determine the most appropriate stress tolerance criteria, the overall grain yield was subjected to a correlation coefficient between yield in irrigated (Yp) and yield in stress (Ys) using the 11 quantitative indices of stress tolerance. This research identified STI and GMP as significant among the screened indices to be used in screening Bambara groundnut, as a positive response was observed with both indices. In addition to other indices, such as GMP and MP that were collectively interpreted as being suggestive of greater drought stress tolerance, Fernandez (1992) had suggested using STI as a measure to distinguish between genotypes with high yield and stress tolerance potentials. Similar studies on 14 soybeans genotype identified GMP and STI as correlating with both stress and non-stress yields, (Kargar et al., 2014). Furthermore, reports by Khodarahmpour et al. (2011) indicated the use of SSI, STI, and GMP indices to be more accurate criteria for selecting high yield and heat tolerance in some genotypes of maize. Research on maize, common bean, barley, and pea by Kristin et al. (1997); Khalili et al. (2004); Karami et al. (2006), and Souri et al. (2005) all proved to identify both STI and GMP were applicable and efficient to separate the different genotypes.

Given that Bambara is extremely cleistogamic, the examined accessions demonstrated significantly greater observed heterozygosity (Ho = 0.5) than what was expected (Ho = 0.01). This high heterogeneity observed among a few of the accessions (up to 20%) may serve as a breeding source for future purposes. Contrary to this, Molosiwa et al. (2015) observed a practically total deficiency of heterozygosity among the individual plants of Bambara groundnut landraces studied, while Odongo et al. (2015) observed an average value of 0.345, which were all lower than what was observed in this study. Blair et al. (2009) also identified a value of 0.64% among the 604 genotypes of common bean analyzed using a set of 36 SSR markers. Likewise, the review by Aliyu et al. (2016) affirmed less than 5% heterozygosity is usually predicted for the self-breeding plant. It is, thus, projected that the population size, possible markers number, and the species type could be key factors in explaining the huge differences observed in the rate of heterozygosity of the studied accessions and can, thus, be explored for subsequent breeding research.

The cluster analysis revealed a distinct pattern of relationships among the 100 accessions included in this study. This was brought about by the mutually exclusive grouping of similar descriptions into the same cluster to authenticate the similar pedigree based on common generated data. The SNPs presented thousands of markers that differentiated the accessions on the basis of geographical origin as it had usually been known with Bambara accessions. The South African accessions stood out distinctively from other accessions from other regions while the West African accessions were found clustering with Central African accessions as earlier evidenced by Olukolu et al. (2012), whose research observed that East Africa accessions did not form a unique cluster but rather clustered with accessions from West Africa. Further supporting the idea that there are multiple centers of diversity and/or domestication for various Bambara species, Aliyu et al. (2016) presented significant evidence for the existence of two primary subpopulations for Bambara groundnut that have developed as separate groups. Previous reports on diversity studies on Bambara had always observed that clustering of genotypes or landraces to be on a known geographical location similar to what was observed in this study, (Amadou et al., 2001; Massawe et al., 2002; Singrün and Schenkel, 2003; Ntundu et al., 2004). This, thus, reaffirmed the possible justification of West Africa being the center of origin of this crop.

The statistical method was another key determinant for the success of the GWAS (Zhang et al., 2015). The MLM is preferred to the GLM model as, by calculating the population structure and the unnecessary relations among the tested individuals, it allows for a massive reduction of false associations. According to the GWAS results of these 100 Bambara accessions, eight SNPs were generated and identified to be significantly associated only with stress-tolerant index (STI) using the MLM model and this is explained by over 80% of the phenotypic variation of the Bambara accessions. The minimum p value of the significant SNPs identified was with markers 24346377, 4177257, 4182070, and 4183904 at (P ≤ 1E-6), while the remaining four markers were at an exponential p value of (P ≤ 1E-4). Similar to what was observed in this research work, Martínez-Ballesta et al. (2015) explained their findings on SNPs that are associated with SSI and STI in relation to the percentage spikelet sterility and yield per plant in rice, which explains a range of 6 – 21% for the phenotypic variation. Edae et al. (2014) also reported one DArT marker on chromosome 4A in wheat and this explained 4% of the phenotypic variation for the Grain Yield-Stress Susceptible Index. Particularly for drought tolerance using DArT markers, Zhang et al. (2017) identified 16 SNP loci and eight candidate genes that were significantly associated while performing a GWAS in a panel of 66 canola accessions, while Schulz et al. (2016) used the GWAS platform to focus on anthocyanin and carotenoid content of petals from 96 diverse rose genotypes. Furthermore, previous studies on Bambara groundnut identified the nearest flanking markers from the pre-selected common marker set for internode length QTL in Bambara groundnut, Ho et al. (2017). All these loci, thus, present a region that could be considered for the development of markers for the subsequent selection of drought tolerance in Bambara groundnut.

Genome-wide association study (GWAS) methodologies applied in this study were able to identify relationships between the SNP markers and stress tolerance index as the phenotypes of interest for the association. The complexity of the trait of interest, known to be controlled by multiple genes, and the critical nature of GWAS facilitated effective and repeated field experiments for different years and different locations. Genome-wide association studies (GWAS) are, thus, valuable tools needed for identifying candidate genes, as well as the genetic loci that are responsible for the variations observed in a targeted quantitative trait.

## Conclusions

In order to feed the continent’s ever-growing population, underutilized crops, such as Bambara groundnut, need an urgent revolution to increase their productivity by utilizing modern technologies. Marker Trait Association is, therefore, a profound key in locating genomic areas linked to these phenotypic traits of breeding importance and, by doing so, can alleviate most of the production constraints. The genome-wide association study on Bambara groundnut was able to establish a strong meeting point between the phenotypic and genotypic traits. With this, it will make it easier to take more steps toward the future exploitation of the identified quantitative loci for marker-assisted selection, marker-assisted backcrossing, and other genomic and breeding initiatives. Future prospects can be improved by conducting an omics study on the identified drought-tolerant accessions to gain a thorough understanding of the genes that can be up- or down-regulated, providing clues as to potential genes that could be introduced for advantageous plant breeding processes for drought stress tolerance.

## Data availability statement

The original contributions presented in the study are included in the article/supplementary material, further inquiries can be directed to the corresponding author/s.

## Author contributions

KO, OAO, and MA conceived and designed the experiments; KO and RP performed the statistical analysis; KO wrote the manuscript and prepared the references; KO, OAO, MA, RP, and OJO, revised the manuscript. All authors contributed to the article and approved the submitted version.
